# Biotechnological Intervention and Secondary Metabolite Production in *Centella asiatica* L.

**DOI:** 10.3390/plants11212928

**Published:** 2022-10-30

**Authors:** Irfan Bashir Ganie, Zishan Ahmad, Anwar Shahzad, Alexandra Zaushintsena, Olga Neverova, Svetlana Ivanova, Adla Wasi, Sabaha Tahseen

**Affiliations:** 1 Plant Biotechnology Laboratory, Department of Botany, Aligarh Muslim University, Aligarh 202002, India; 2 Co-Innovation Center for Sustainable Forestry in Southern China, Nanjing Forestry University, Nanjing 210037, China; 3 Bamboo Research Institute, Nanjing Forestry University, Nanjing 210037, China; 4 Research Institute of Biotechnology, Kemerovo State University, Krasnaya Street 6, Kemerovo 650043, Russia; 5 Department of Ecology and Nature Management, Kemerovo State University, Krasnaya Street 6, Kemerovo 650043, Russia; 6 Natural Nutraceutical Biotesting Laboratory, Kemerovo State University, Krasnaya Street 6, Kemerovo 650043, Russia; 7 Department of General Mathematics and Informatics, Kemerovo State University, Krasnaya Street, 6, Kemerovo 650043, Russia

**Keywords:** *Centella asiatica*, elicitation, hairy root culture, micropropagation, synthetic seeds, triterpenoids

## Abstract

*Centella asiatica* L., commonly known as Gotu kola, Indian pennywort, and Asiatic pennyworts, is an herbaceous perennial plant that belongs to the family Apiaceae and has long been used in the traditional medicine system. The plant is known to produce a wide range of active metabolites such as triterpenoids including asiatic acid, asiaticoside, brahmoside, and madecassic acid along with other constituents including centellose, centelloside, and madecassoside, etc., which show immense pharmacological activity. Due to its beneficial role in neuroprotection activity, the plant has been considered as a brain tonic. However, limited cultivation, poor seed viability with low germination rate, and overexploitation for decades have led to severe depletion and threatened its wild stocks. The present review aimed to provide up-to-date information on biotechnological tools applied to this endangered medicinal plant for its in vitro propagation, direct or indirect regeneration, synthetic seed production, strategies for secondary metabolite productions including different elicitors. In addition, a proposed mechanism for the biosynthesis of triterpenoids is also discussed.

## 1. Introduction

*Centella asiatica* (L.) Urban (Family–Apiaceae), commonly known as Gotu kola, is a centuries-old traditional herb used in Southeast Asian countries and China to treat different diseases. The earliest records suggest that *C. asiatica* was used during the Song Dynasty of China [1]. According to a Chinese literature source, the plant is described as “ming jia fang xuan” which means “the herb has been used for the treatment of Gilles de la Tourette syndrome in children’’ [1]. *C. asiatica* is believed to be the most prevalent species in the areas of South Africa, China, Sri Lanka, Colombia, Venezuela, Mexico, and eastern regions of South America.

Other representatives of *C. asiatica* are found in various regions of South Africa [2,3]. In India, the plant grows at a height of 1200 m above sea level in the parts of Rajasthan and Sikkim [4]. The plant grows in areas with high humidity and moist soil. Species like *C. asiatica* and *C. glabrata* are mostly used for conventional treatment in South Africa [5]. Traditionally, the medicinal plants have been the source of important pharmaceutical compounds which are greatly utilized by various pharmaceutical as well as cosmetic industries for the preparation of medicinal and cosmetic formulations. Asian countries have been using *C. asiatica* over centuries to treat different kinds of diseases [6]. In Madagascar, *Centella asiatica* is collected by native people to fulfill both their social and economic interests alongside exporting the herb in greater quantity to meet the growing demand of pharmaceutical and cosmetic companies [7]. In Asian countries, *C. asiatica* is used as an essential ingredient in the traditional system of medicine often called Ayurveda and several reports claimed that *C. asiatica* possesses remarkable protective effect against several diseases affecting the central nervous system of humans [8]. *C. asiatica* is reported to possess diverse medicinal effects including neuroprotective, antidepressant, cardioprotective, anticancer, antimicrobial, anti-inflammatory, gastroprotective, and antioxidant properties [9,10]. A detailed description of its medicinal use can be found in the next section. The medicinal properties of *C. asiatica* have been attributed to its richness in biologically active compounds such as asiatic acid, asiaticoside, madecassic acid, and madecassoside (Table 1) [11]. 

A survey conducted by Nayar and Sastry [14] reported the shocking decline of the wild population of *C. asiatica* due to unchecked and unregulated exploitation of the herb, particularly for medicinal purposes, coupled with the absence of efforts for its organized cultivation. Due to a gradual decline in its numbers, its limited cultivation, poor seed viability, and insignificant restoration efforts, the herb has been included in the threatened category by the International Union for Conservation of Nature and Natural Resources (IUCN) and has also been categorized as an endemic species to the Western Ghats of Southern India [15]. Over the years, there has been a growing interest in plant tissue culture or in vitro culture studies which offer a viable alternative for the propagation and conservation of endangered medicinal plants [16]. Therefore, employing the in vitro techniques for the propagation of *C. asiatica* on a large scale, particularly of its superior clone, will contribute to the restoration of its population. The proposed review deals with biotechnological approaches applied to *C. asiatica*. The study reveals the strategy of obtaining biologically active substances using the in vitro method and also discusses the mechanisms of triterpenoid biosynthesis.

## 2. Traditional and Medicinal Use

Due to its great medicinal properties, *C. asiatica* has been used against a number of diseases for centuries (Table 2) [17]. *C. asiatica* leaf powder is often consumed in India to revitalize the nerve and brain cells, and therefore, it is often known as Brain Food [18]. The syrup made from the mixture of *Centella* leaf extract, black pepper, and ginger is used to treat cough and bone fractures [19]. Similarly, a paste made from fresh leaves of *Centella* is applied to boils. Leaf syrup made of *Centella* is used to relieve headache pain by smearing the syrup on the forehead [20]. In fact, the whole plant has been used to treat diseases such as stomach disorders, asthma, leprosy, urethritis, dog bites, and tumors [21,22]. Similarly, in Brazil, *C. asiatica* leaves are used for the treatment of elephantiasis [21]. Interestingly, people in Malaysia use the plant to prepare a salad commonly known there as “Ulam”. The dried powder of *Centella* is used for tea preparation and the fresh leaves for juice preparation in many East Asian countries [23,24].

*Centella asiatica* contains triterpenes which are reported to be the main compounds that show biological activity [40,41]. Triterpenes such as asiaticoside, madecassic acid, madecassoside, and asiatic acid are the principal compounds that are believed to be responsible for various medicinal activities of a plant. Studies reported that plant extract and asiatic acid enhance the learning and memory potential, while other studies reported that the antioxidant potential contributes to increasing cognitive potential [42]. Similarly, the plant extract contains amyloid beta (Aß) levels responsible for Alzheimer’s disease (AD) [43]. Furthermore, one of the studies reported that asiatic acid downregulates beta-secretase 1 (BACE1) which further reduces the Aβ level [44]. Asiaticoside and madecassoside are the two main compounds of *C. asiatica* that have shown anti-inflammatory activity [45]. Similarly, asiaticoside has been reported to treat dementia in elderly people. Reactive oxygen species have been reported to play an important role in causing aging in humans. The higher production of ROS coupled with a weak defense mechanism leads to cell death and aging-related complications such as weak memory, dementia, etc. [46]. A great deal of research on the regenerative potential of *C. asiatica* has been conducted on the central nervous system (CNS). Due to the increased antioxidant activity, the aqueous extract of *C. asiatica* has shown neuroprotection in the nervous system of mice [47]. Similarly, the use of solvents to produce extracts of *C. asiatica* such as ethyl acetate, *n*-butanol, ethyl acetate, *n*-hexane, and chloroform has shown significant levels of neuroprotection in albino rats [48]. Extract from the fresh leaves of *C. asiatica* has shown significant enhancement in the memory and learning process when investigated on neurons of adult rats [49]. However, no significant breakthrough has been reported that confirms the possible mechanism of neuroprotection induced by centellosides of *C. asiatica*. However, certain reports have suggested that MEK/ERK, P13/Akt pathways are believed to be involved in neuroprotection triggered by centellosides of *C. asiatica* [50]. One of the findings reported is that asiatic acid has shown neuro-regeneration activity via the MAP kinase pathway [51]. Similarly, Wanakhachornkrai et al. [52] reported that ECa 233 from *C. asiatica* promotes elongation of neurites via MEK/ERK- and PI3K/AKT- pathways. Omar et al. [53] reported that *C. asiatica* regulates the apoptotic pathways in neuron cells and, therefore, protects them from undergoing unnecessary apoptosis. They reported that l-buthionine- (S,R)-sulfoximine (BSO)-treated human neuron cells survived when treated with an ethanolic extract of *C. asiatica.* The authors concluded that at low concentrations, the ethanolic extract significantly inhibits the oxidative stress responsible for damaging the neuron cells and causing the activation of proapoptotic proteins. 

## 3. Micropropagation

Micropropagation is one of the best and efficient alternatives for the conservation of endangered medicinal plants [41]. Several medicinal plants have been conserved by using the techniques of micropropagation. It is an extraordinary approach for the rapid propagation of a plant in a controlled environment. Extensive research has been conducted on the in vitro propagation of *C. asciatica* and many reports have been published. In this section, the micropropagation of *C. asciatica* is summarized [42]. 

**Figure 1:**
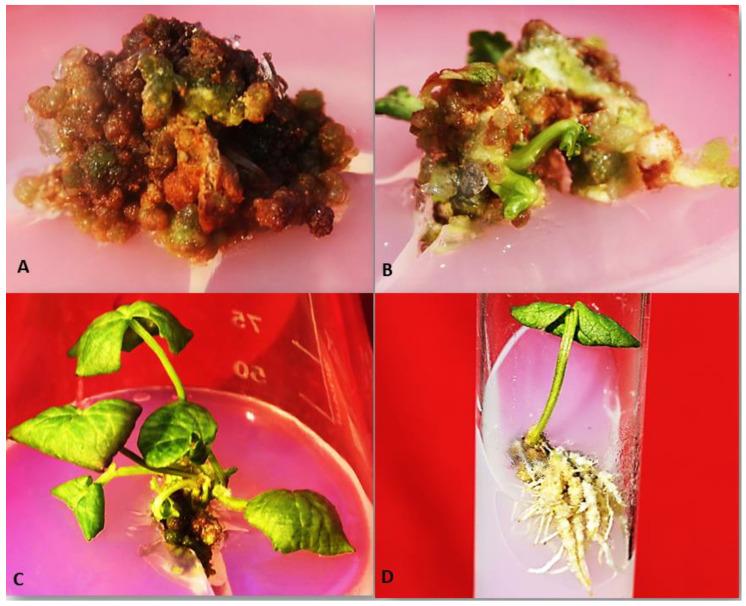
(**A**) Callus formation on the 12th day of inoculation on MS media augmented with BA (6 μM) + NAA (0.8 μM). (**B**) Multiple shoot initiation from callus cultures of *C. asiatica* in the first week of subculture on MS media augmented with BA (8 μM) + KN (0.6 μM). (**C**) Shoot multiplication at the 2nd passage of subculture on MS media augmented with BA (8 μM) + KN (0.6 μM). (**D**) Regeneration of roots from shoots of *C. asiatica* cultured on MS medium augmented with NAA 1.5 μM. (Unpublished photographs of Dr. Anwar Shahzad).

In experimental studies [54], the formation of calli on the cut surfaces of the *C. asciatica* explant in various nutrient media was studied. For callus induction and morphogenesis, nodal segments (1–1.5 cm) were cultured on MS medium augmented with various cytokinins and auxins. The highest amount of callus proliferation was obtained on MS medium comprising BA (6 µM) + NAA (0.8 µM) and 2,4-D (2.5 µM) with 95 and 87% response, respectively. The calli obtained from MS medium augmented with BA (6 µM) + NAA (0.8 µM) were subcultured on a regeneration media. Among all the treatments evaluated, MS + BA (8 µM) + Kn (0.6 µM) and MS + BA (8 µM) + NAA (0.5 µM) established the best responses with the production of 12.2 and 8.2 shoots per explant with 80.4% and 62% response, respectively, within 6 weeks of culture. For root induction, microshoots of 4–5 cm in length were excised and planted onto the rooting medium composed of half supplied MS medium augmented with various concentrations of NAA and IBA. The best rooting response (92%) was observed on MS medium augmented with NAA (1 µM) with an average 8 roots, followed by 67% on half supplied MS medium augmented by IBA (0.5 µM) with an average 5 roots developed per shoot. The rooted plantlets were transferred to earthen pots containing soil: soilrite in the ratio of 1:1:1 and acclimatized successfully therein. Figure 1 presents the micropropagation of *C. asciatica* from our own unpublished experimental work.

The study [55] showed that on *C. asiatica* leaf explants, callus was formed on the cut surfaces, which subsequently covered the entire surface of the explant within 15–20 days. Media containing a low concentration of naphthaleneacetic acid (NAA; 2.68 μM) or a low/high concentration of 6-benzyladenine (BA; 2.21 and 17.68 μM) did not contribute to the induction of callus formation. Green, compact, and embryogenic callus had a 66–100% response at different concentrations of BA and NAA. The efficiency of callus formation was 85%, the efficiency of shoot formation was 90%. However, Patra et al. [56] reported that the addition of NAA to an MS medium containing BA or kinetin reduces the callus formation reaction. While the best callogenic response of leaf explants was observed on MS medium supplemented with BA or kinetin together with 2,4-dichlorophenoxyacetic acid, good callus formation was not observed when the media were supplemented with BA and NAA [57]. 

### 3.1. Direct Propagation

#### 3.1.1. Nodal Culture

The composition of the media, plant growth regulators (PGR), and culture conditions greatly influence the micropropagation of plants. Therefore, all culture inputs, particularly PGR concentrations, are optimized to study the in vitro morphogenetic response of various plant species. A description of the explant sterilization procedure in *C. asciatica* has been given in Table 3. Tiwari et al. [58] reported that nodal segments, when inoculated on MS media supplemented with 5 mg/L BAP and 0.5 mg/L NAA, show the best shoot multiplication. In another report, an efficient two-step propagation of *C. asiatica* was conducted by Tiwari et al. [59] in which they reported that BA at 4 mg/L and NAA at 0.5 mg/L of concentration supplied with MS media significantly increases the number of shoots per explant and MS media supplemented with 4 mg/L BA and 0.4 mg/L NAA induced a higher rate of shoot multiplication. Prasad et al. [60] conducted a study to find out the effect of fungal elicitors on the shoot cultures of *C. asiatica*. They took 1–4% (*v*/*v*) culture filtrate of *Trichoderma harzianumin* and added it into the medium on the 0th, 10th, and 20th day of the culture cycle. It was observed that it did not affect the growth of the shoots. Instead, the cultures treated with 3% culture filtrate resulted in a 1.24-fold increase in shoot growth, besides increasing the growth index (GI) of the plant. The nodal cultures of *C. asiatica* inoculated on MS medium devoid of PGR failed to show any growth response. However, MS media augmented with BA alone or in combination with NAA induced axillary shoot proliferation. The growth rate was reported to increase with consistent increase in BA concentration up to an optimum 22.2 μM BA concentration. Furthermore, the combination of BA and NAA increasing the frequency of axillary shoot proliferation with maximum shoot frequency (91%) was attained with MS media augmented with 22.2 μM BA and 2.68 μM NAA. However, callus formation took place when the concentration of NAA was increased beyond 2.68 μM. Previously, Banerjee et al. [61] reported that BA and NAA at higher concentration resulted in callus formation from leaf explants in *C. asiatica*. Table 4 describes the developed direct propagation protocols on *C. asiatica.*

When only leaves are sterilized [57], up to 7–8 shoots are formed; when only nodes are sterilized [59], a small number of shoots are also formed: up to 8; when leaves and stems are sterilized [65,66], up to 10 shoots are formed. The largest number of shoots (10–13) is formed during sterilization of nodal explants [69,70]. Thus, the procedure for fast and effective sterilization of nodal explants is as follows: RTW (30 min) → 1% teepol (5 min) → 0.1% HgCl _2_ + teepol (3 min) → 3–4 × SDW → 0.1% HgCl _2_ (3 min) → SDW [70].

Similarly, Nath and Buragohain [62] observed that BA alone or in combination with KN and NAA promotes shoot regeneration of *C. asiatica.* Furthermore, they reported that BA alone promotes a lesser number of shoots from a single shoot tip explant as compared to MS medium augmented with BA (4.0 mg/L) and NAA (0.1 mg/L). However, further increase leads to the callus formation as reported by Banerjee et al. [60]. The superiority of BA over KN in inducing multiple shoot regeneration has been reported earlier in a number of medicinal plants [73]. The optimum number of shoots (3.38) and leaves (4.25) per explant was obtained on MS augmented with 4.0 mg/L BA and 0.1 mg/L NAA. It has further been reported that the percentage of shoot development reached the highest of 90%: four to five shoots per node when the nodal segments excised from primary shoot cultures were inoculated on MS medium supplemented with 22.2 μM BA and 2.68 μM NAA. However, callusing at the base of cultures has been reported when the cultures were passed through three subculture passages. From time to time, efforts have been put forward to provide an efficient and easy to handle protocol for the regeneration of *C. asiatica* on a large scale. Singh et al. [63] reported that nodal segments inoculated on MS media augmented with BA (2 mg/L) and KN (0.5 mg/L) led to an increased number of shoot numbers (16.3) per explant. Similarly, cefotaxime, bavistin, and kanamycin which are categorized as antimicrobials have also been augmented with MS to study the shoot-promoting effect. It was observed that among the three antimicrobials studied, the highest number of shoots, i.e., 6.6 per nodal explant were produced from MS media augmented with BA (2 mg/L) and bavistin (150 mg/L) [64,72].

#### 3.1.2. Bud Breaking

The multiplication of shoots through bud breaking is another useful method for the enhanced multiplication of desired germplasm. Tiwari et al. [59] reported that studying the morphogenetic response of a nodal culture would be better when plant growth regulators were added to the nutrient medium. The early bud breaking response is a rational strategy to pursue the fast and time consuming regeneration of a plant. Various combinations were tested for early bud break in *C. asiatica,* and reports have claimed that BAP (2 mg/L) + GA3 (0.5 mg/L) showed the early bud breaking response. Bhandar et al. [72] studied the effect of various PGR combinations on bud breaking in *C. asiatica*. They observed that the BAP (0.5 mg/L) + NAA (0.5 mg/L) and BAP (0.1 mg/L) + adenine (0.5 mg/L) combinations took 84 h for bud breaking which was a very long period. However, BA alone at a concentration of 2 mg/L induced early bud breaking. Similar results were obtained in *Ocimum basilicum* by Pattnaik and Chand [74]. In addition, BAP has also been reported to induce shoot proliferation in a number of species [75]. BAP in combination with GA3 at different concentrations has significantly caused the early bud breaking and enhanced the percentage of shoot proliferation. Sharma and Sharma [76] reasoned that early bud breaking was due to the movement of hydrolytic enzymes and their stimulating effect. However, Tiwari et al. [59] reported that a high concentration of BAP (5 mg/L) improved bud breaking, and when used along with IBA (0.5 mg/L), enhanced root growth was observed. Karivartharaju and Ramakrishnan [77] described that the shoot elongation might be due to the cell enlargement and increased cell division when the cultures are inoculated on a medium supplemented with BAP and GA_3_. 

#### 3.1.3. Shoot Tip Culture

Shoot tip culture is an effective in vitro approach to develop disease-free plants on a large scale. Sivakumar et al. [78] reported that a shoot tip segment of *C. asiatica* successfully induced higher frequency of bud break (88%) followed by multiple shoot formation when cultured on MS medium augmented with 6-benzylaminopurine (BAP) (17.76 µm) and gibberillic acid (GA_3_) (1.44 μM). Bud break and the development of multiple shoots from shoot tip culture is due to the reduced application of cytokinins concentration. The shoot tip explants cultivation did not evoke any response even after 30 days of inoculation. However, MS medium augmented with BA has induced bud break and multiple shoot induction. There has been positive correlation between the BAP concentration and shoot regeneration, as it was reported that with an increased concentration of BAP, the multiple shooting response also increased till an optimum concentration (17.76 μM) was achieved at which about 80% of bud break was observed in shoot tip culture of *C. asiatica.* The first sign of response the shoot tip showed was the enlargement of the existing shoot bud followed by a bud break within a time period of 8–19 days. In succeeding the primary event, the shoot bud developed a single shoot within 12–15 days. Thereafter, new shoots (9–10 shoots/bud) with an average height of 5.64 cm were formed adjacent to the primary one within a time period of 20–25 days. It was reported that with the increase in the concentration of BA beyond 17.76 µM—an optimum concentration—a decrease in the bud break and multiple shoot induction was observed. The stimulatory effect of BA alone in *C. asiatica* was also reported in *Solanum trilobatum* and *Gloriosa superba* [79]. If the concentration exceeded the optimum concentration of BA, the bud breaking was suppressed significantly. 

Similarly, the use of GA_3_ singly in shoot tip cultures has resulted in an increase in sprouting. However, the bud break and multiple shoot induction were reduced significantly. A maximum of 15–20% of shoot buds sprouted within a time period of 25–30 days when the shoot tips were inoculated on MS medium inoculated with an optimal concentration of GA_3_ (1.44 μM). Any concentration beyond 1.44 μM was observed to have no promotive effect on multiple shoot development. Instead, a progressive increase in the shoot length was observed. A combination of BAP (17.76 μM) and GA_3_ (1.44 μM) in MS culture medium has shown a synergistic effect by causing both an increase in bud break (16.8 shoots/shoot tip) and a higher frequency in multiple shoots induction.

### 3.2. Indirect Organogenesis

#### 3.2.1. Callus Formation

The callus induction is a first-stage somatic embryogenesis. Various PGRs have been used for the callus induction of *C. asiatica.* Auxins such as 2,4-dichlorophenoxyacetic acid (2,4-D), Picloram, and Dicamba have shown a positive response for the induction of calli. However, the rate of callus growth, color, and fresh weight were markedly different in both petiole and leaf explants of *C. asiatica*. It has been reported that all the above-mentioned auxins successfully induced callus formation, and it has been observed that the callus formation increased with an increase in 2,4-D concentrations till the concentration reached 6 mg/L, at which point the callus induction percentage reduced significantly. Dicamba has rarely been used in tissue culture studies; however, it has been reported that Dicamba is effective when it comes to callus induction in *C. asiatica*. It was reported that all the concentrations of Dicamba have shown 100% callus formation with the highest fresh weight percentage on the medium containing 4 mg/L Dicamba, i.e., 0.27 g. Similarly, 2,4-D has shown 100% callus induction on the 6th week of culture when the leaf explants were inoculated on MS augmented with 2 and 4 mg/L–2,4-D, and beyond 4 mg/L–2,4-D, the percentage of callus induction reduced to 81.3%. It has been reported that petiole cultures of *C. asiatica* have shown 100% callus formation when inoculated on MS media augmented with 2,4-D and Dicamba; however, the highest percentage of callus growth and fresh weight has been obtained at 4 mg/L 2,4-D and Dicamba. Similarly, Patra et al. [56] reported that MS media augmented with 2.0 mg/L KN and 4.0 mg/L NAA induces callus formation from leaf and stem cuttings of *C. asiatica*, while Martin [57] induced callus formation on MS medium augmented with 2,4-D and NAA (1.0 mg/L) in combination with 0.5 mg/L KN. Moreover, Loc and Tam [80] observed that MS media augmented with BA (1.0 mg/L) and NAA (1.0 mg/L) produced compact and friable calli after 3 weeks of petiole culture. More studies have revealed that MS medium with no plant growth regulator supply has failed to induce callusing in any explant of *C. asiatica*. Moreover, 2,4-D at a concentration of 2.0 mg/L has shown a significant effect for increasing the rate of callus initiation, and BA at a concentration of 4.0 mg/L was the most effective concentration for mass induction. Rao et al. [81] observed that NAA (2.0 mg/L) in combination with KN (0.2 mg/L) induced callus from the leaf explants of *C. asiatica.*

#### 3.2.2. Shoot Regeneration

Shoot development from any explant depends on the type of media composition supplied with cytokinins as plant growth regulators. Most of the protocols for shoot development in *C. asiatica* have used basal media for micropropagation. MS medium supplemented with BAP (3 mg/L) and NAA (0.05 mg/L) was used for shoot development in leaf explants of the plant [59]. Similarly, Hossain et al. [82] achieved the highest shoot regeneration frequency at BAP 1 mg/L and NAA 0.5 mg/L from the nodal explants. Naidu et al. [83] reported that two types of calli (embryogenic and nonembryogenic) were developed from nodal segments when inoculated on a medium supplemented with auxin concentration followed by the light and dark cycle of exposure. They further observed that a white embryonic structure was developed on the surface of the callus and was categorized as embryonic which has a potential to develop into various stages of embryoids. On the other hand, those that were yellow, soft, and lacking proembryoid structures were categorized as nonembryogenic calli. Each embryogenic callus usually continued to shoot regeneration, while the nonembryogenic type turned brown and stopped growing further. It was observed that the maximum shoot percentage was reported from calli of leaf and stem cuttings when inoculated on MS media augmented with 4 mg/L BA, 2 mg/L KN, 0.25 mg/L NAA, and 20 mg/L Ads (Adenine sulfate) [62]. Similarly, Naidu et al. [83] reported the highest percentage of shoot induction from calli when transferred to MS medium supplemented with 1.5 mg/L BA and 1.5 mg/L KN (Table 5) [55].

### 3.3. Somatic Embryogenesis

The intrinsic plasticity of plants, which is a unique attribute among plants, allows plant species to adapt to various environmental changes. In vitro culture uses this feature of the plant to control the particular morphogenetic response of the plant. The morphogenetic responses of the plant under in vitro conditions include organogenesis, somatic embryogenesis (SE), androgenesis, gynogenesis, and micropropgation. Somatic embryogenesis is considered to be one of the best morphogenetic responses that raises true to type plants on a large scale [86]. Somatic embryogenesis is a process that involves the formation of embryogenic cells from somatic cells under in vitro conditions. The embryogenic cells, upon various morphological and biochemical changes, result in the formation of somatic embryos [87]. Somatic embryogenesis is considered as one of the important techniques of plant biotechnology with the potential to bring about the desired genetic changes in the somatic embryos for genetic improvement of a particular species, besides transforming them into artificial seeds for broader applications of biotechnology for human interest [88]. Plant regeneration, particularly through shoot morphogenesis, occasionally results in genetically variable and chimeric plants. However, in the case of somatic embryogenesis, the plants are genetically similar whether the somatic embryos are directly or indirectly produced [89]. Reports suggested that auxin (2,4-dichlorophenoxyacetic acid, indole acetic acid) acts as a trigger molecule for the induction of somatic embryogenesis. Other reports suggested that auxin in combination with cytokinins plays a vital role in inducing somatic embryogenesis [90]. However, certain reports suggested that the use of unconventional plant growth regulators (PGRs) such as thidiazuron has also shown the induction of somatic embryogenesis in the hypocotyl cultures of geranium, sugar beet, and *Paulownia elongate* [91]. In addition, the use of additives such as glutamine, proline, casein hydrolysate, and sugar in higher amounts positively influences the somatic embryogenesis. It was further reported that in *C. asiatica,* cultures of stolon and leaf explants have successfully induced somatic embryogenesis when allowed to culture on MS media augmented with PGRs. However, the stolon responded via direct route and the leaf via the indirect mode of somatic embryogensis which involves an intervention of the callus stage before the induction of somatic embryos [92]. In one of the studies, somatic embryos developed on medium augmented with 9.29 μM kinetin in combination with 2.26 μM 2,4-D indicating the central role 2,4-D plays in this process [92].

#### Indirect Somatic Embryogenesis

The indirect somatic embryogenesis involves the intervening callus stage followed by the induction of somatic embryos. The leaf and intermodal explants of *Centella asiatica* have shown the callus induction on MS medium augmented with NAA, IAA or BA in combination with NAA, and 2,4-D in combination with NAA. Callus texture and type of callus depend on the type and concentration of growth regulators [57]; MS medium augmented with 4.52 μM 2,4-dichlorophenoxyacetic acid or 5.37 μM a-naphthaleneacetic acid (NAA), both with 2.32 μM kinetin (KN), was reported to induce somatic embryogenesis. Furthermore, calli developed on medium supplemented with NAA and KN favored induction and maturation of embryos compared to calli developed on MS medium augmented with 2,4-D and KN. Embryogenic calli transferred from MS medium augmented with NAA (2.69 μM) and KN (1.16 μM) developed 204.3 mean number of somatic embryos when transferred into a suspension culture augmented with half-strength MS medium with NAA (2.69 μM) and KN (1.16 μM). Furthermore, 88% of the embryos matured into plantlets upon their transfer to half-strength MS semisolid medium having 0.054 μM NAA with either 0.044 μM BA or 0.046 μM KN [57]. Similarly, leaf segments of *C. asiatica* were observed to produce a large number of somatic embryos when allowed to culture on MS medium augmented with 9.29 μM kinetin in combination with 2.26 μM 2,4-D. After one week of culture, shiny, granular, and white calli were developed which further turned into other developing stages such as heart-shaped and cotyledonary stage when cultured on the same medium for 4 weeks. The somatic embryos successfully germinated on MS medium augmented with 2.32 μM kinetin and GA_3_ (2.89 μM) [57].

BAP at higher concentrations, 4.44 and 8.87 µm in combination with 2,4-D at 0.45 and 2.26 μM, develops embryogenic calli. Furthermore, BAP at higher concentrations in combination with KN at lower concentrations has also resulted in embryogenic calli. However, BAP at 8.87 μM concentration in combination with 2,4-D at 2.26 μM was the most effective concentration which resulted in 84% of somatic embryo induction with 37.3 average somatic embryos per explant [92]. Samataray et al. [93] reported the induction of somatic embryogenesis in *Echinochloa colona* by cytokinin in combination with auxin. Similarly, the induction of somatic embryos by cytokinin + auxin combination has also been observed in *Dendanthema grandiflorum* and *Bacopa monnieri* [94,95]. The somatic embryos took three weeks to develop into complete embryos from the abaxial surface of the leaf.

Kunta and Mani [96] used various explants of *C. asiatica* such as nodal segments, leaf, and petioles for the induction of somatic embryos and various concentrations of auxins and cytokinins were tried. Of the various combinations tried, 2,4-D + KN and 2,4-D + IAA were found to be the most effective combinations for somatic embryo induction. KN at higher concentrations (3 mg/L) in combination with 2,4-D at 2 mg/L resulted in 84% of embryogenic calli formation. Within the first week of culture, greater granular and shiny masses of calli were produced from the abaxial surface of the leaf petiole region, nodal segments, and leaf of the herb. Later in the second week, the callus mass turned globular and light green in color followed by heart- and torpedo-like structures in the third and fourth week of culture, respectively. Sucrose has been observed to have a role in influencing the induction of somatic embryogenesis, and it has been observed that sucrose at 4% was found to be an effective concentration in *C. asiatica*. However, sucrose at 1–3% failed to show any positive response for somatic embryogenesis. It is widely believed that sucrose serves as a source of carbon and, therefore, it must have a role in the organization and maturation of somatic embryos. Table 6 represents the developed indirect propagation protocol of *C. asiatica.*

### 3.4. Rhizogenesis

The shoots excised from parent shoot culture failed to induce rooting in plant growth regulator-free MS medium. Of the three auxins tested on rooting in *C. asiatica*, however, NAA (10.74 μM) was observed to be the most effective. About 90% rooting was developed at an optimal concentration of 10.74 μM with 19 roots per shoot within a period of 12–15 days on half-strength MS medium supplemented with NAA. Upon 30 days of culture in the same medium, the roots attained a length of 4.9 cm with 25–28 roots per shoot. Ex vitro rooting is an efficient strategy. According to Debergh and Maene [97], ex vitro rooting reduces by about 40–75% the cost of the regenerated plants. Moreover, it saves on the expensive use of culture conditions of the growth room. The ex vitro roots of the plants had no difference from the in vitro rooting besides maintaining the growth parameters of the plantlets during their transfer. More importantly, all the plantlets developed exhibit morphological similarity with the mother plants. Ex vitro rooting has also been reported in *Gardenia jasminoides* and *Veronica* [98]. In the case of *C. asiatica*, the basal portion of shoots were dipped in various concentrations of IBA for about 10 days. Among the various concentrations of IBA tested, 9.84 μM of IBA was found to be the most effective as the roots started to induce within a time period of only 10 days. Moreover, an average of 30.32 roots/shoot were developed in the IBA solution. The rooting induced through this method has shown 100% survival upon their transplantation. Table 6 represents the developed protocol in *C. asiatica.*

### 3.5. Synthetic-Seed Technology

Seeds are an outcome of sexual reproduction in cross-pollinating plants; however, the seeds produced by this natural phenomenon are most likely to be genetically dissimilar from the parental stock [99]. The ability of seeds to undergo dormancy for a particular period of time or until supported by their required growth conditions enables the seeds to be stored and preserved for longer periods of time. The plants picked up from their natural habitats and distributed across continents could become a source of disease dissemination, as natural conditions often invite pathogens [100]. Synthetic seed technology has gained attention due to its potential to preserve the germplasm of a particular plant besides allowing their safe storage and dissemination [101]. More importantly, the syn-seed technology has a vital role in the mass propagation of elite plant species where sexual reproduction does not take place. These encapsulated (Figure 1) embryos can be used for the conservation of important medicinal plants besides facilitating the exchange of materials across world research centers [102,103].

The successful production of synthetic seeds containing embryos or any other vegetative plant part can open new vistas in medicinal and floriculture sectors. The application of artificial seeds depends mainly on the quality, i.e., morphology, biochemistry, and physiology of somatic embryos [104,105]. Since the first claim laid by Steward et al. [106], somatic embryogenesis has been reported in a large number of tropical plants such as cereals, fruits, fibers, legumes, beverages, etc.; however, in most of the cases, the percentage of embryo conversion into plantlet has not been significant [105]. For commercial purposes and for the success of artificial seed technology, large-scale, high quality embryo generation of some important medicinal plants is absolutely necessary. The percentage of sodium alginate greatly influences the syn-seed and the morphological attributes of the plant that germinates from that seed. However, sodium alginate concentration cannot be universally applied on every plant species, optimization of sodium alginate concentration must be given priority for obtaining a better quality of seeds [107]. This is because alginate, being a natural product derived from the cell wall of the brown algae *Macrocystis* and *Laminaria*, often shows batch to batch inconsistency in its geling properties [105]. Therefore, the synthetic seed technology allows the large-scale conservation of important medicinal plants at lower temperature for longer durations, and this feature of this technology allows the swift exchange of seeds for commercial purposes and for medicinal use.

### 3.6. Acclimatization

The direct exposure of micropropagated plants to the external atmosphere would result in the death of all or a maximum number of plants due to their sudden transplantation and lower resilience capability towards the septic environment with high light intensity and low relative humidity. Therefore, plants are transplanted to the external environment in a sequential and gradual manner until the plants get accustomed to in vivo conditions, therefore, ensuring the survival of a maximum number of plants. Patra et al. [56] transferred rooted plantlets into pots containing cow manure, soil, and sand in the ratio of 1:1:1. Moreover, the plants were augmented with MS solution once, one week prior to their transfer into the greenhouse on their 14th day of exposure. The survival rate among plants was reported to be about 55–56% with no morphological alterations. Similarly, Sivakumar et al. [108] transferred rooted plantlets into a tray containing a soil mixture of vermiculite, peat moss, and perlite, and the tray was kept in a growth chamber for about 2 weeks. After 2 weeks of growth, the plantlets were transplanted into the glasshouse and, eventually, were planted into pots containing natural red soil. The plants have shown a 90% survival rate once transferred into the natural environment with zero morphological variations when compared with the mother plant. Furthermore, plantlets derived from somatic embryos that were transferred to pots filled with sand and soil in the ratio of 1:1 have shown good growth and strength within the second week of transplantation. Upon their further transplantation into an open field, the regenerated plants have shown good survival rate with all plants exhibiting a morphological similarity with their mother plant [57].

## 4. Strategies to Enhance Secondary Metabolites

The secondary metabolites (SM) play an important role in aiding the plant to adapt to myriad forms of environmental changes besides being a source of some of the essential pharmaceuticals [109]. However, it has been reported that a majority of the plants produce SM in small quantities; however, their role in treating various disease disorders could be limitless. Therefore, in vitro studies have provided an alternative approach for the enhancement of SM and one such approach includes suspension culture [110]. Plant suspension culture has often been used on a large scale for the production of secondary metabolites. In addition, elicitation has been another in vitro strategy whereby the use of chemical and biological elicitors is allowed to be augmented in the medium for the enhancement of SM [109].

### 4.1. Callus and Cell Cultures

Several researchers have established cell and suspension cultures which have shown an efficient alternative for obtaining centellosides, albeit at a medium scale. In 1993, Solet [111] came up with the first report that reported that a significant amount of centellosides could be enhanced from calli and suspension cultures of *C. asiatica*. It has been reported that asiaticoside production significantly dropped after augmenting the B5 liquid medium with a range of auxin and cytokinin hormones. However, 0.025 mg/L TDZ has been the only concentration that has shown enhancement in asiaticoside production [112]. Similarly, the centelloside enhancement was studied in calli and cell cultures of two phenotypically different South African *C. asiatica.* The total centelloside levels were obtained in higher values in calli (1.5–1.8 times) followed by cell suspension cultures. However, the fact of the matter is that centelloside production was obtained in lower amounts in the calli as compared to the differentiated parts of the plant. The optimization of callus cultures of *C. asiatica* has revealed that main phytocompounds such as madecassoside, asiaticoside, madecassic acid, and asiatic acid could have a total production of 900 µg/g DW. The lower expression levels of some of the key genes such as CabAS and CaSQS genes that are involved in the biosynthesis of triterpenes in calli compared to the leaves of plant explains the lower amounts of centelloside production in calli and callus cultures [113].

### 4.2. Elicitation-mediated Enhancement in Triterpenoids in Centella asiatica

Elicitors can be biological or chemical products produced by the plants under stress. Therefore, exogenous application of elicitors to in vitro cultures could be used to study the response of plant cells towards a potential microbe or any chemical substance. The effect of some of the elicitors such as yeast extract (YE), methyl jasmonate (MJ), salicylic acid, pectin, chitin, and chitosan on the enhancement of secondary metabolites of medicinally important plants has been investigated frequently. It has been reported that SA increases the phenolic compounds in in vitro cultures of *Matricaria chamomilla* [114]. However, the molecular mechanism of the genes involved in the enhancement/synthesis of phenolic compounds is yet to be known. Phillips et al. [115] observed a severe accumulation of rosmarinic acid in the cultures of *Lithospermum erythrorhizon* elicited by YE.

#### 4.2.1. Abiotic Elicitors

##### Salicylic Acid

Salicylic acid is a signaling compound in plants that is reported to activate various defense-related genes when used as an elicitor in the medium. It has been reported that SA is often used for investigating the accumulation/enhancement of pathogenesis-related phytocompounds [116]. In one of the studies, it was reported that SA at 50–200 µm concentration on the day of inoculation followed by 100 µm on the 5th–15th day significantly influences the growth and asiaticoside content in the suspension culture of *C. asiatica.* It was observed that while the biomass of cultures reduced significantly vs. control (0.24–0.42 g of dry mass vs. 0.57 g of dry mass with respective GIs of 1.20–1.87 and 2.01), the asiaticoside content greatly increased in the culture (46.93–102.63 mg g^−1^ of dry mass vs. 46.58 mg g^−1^ of dry mass), and on the 10th day of inoculation, the asiaticoside level reached its highest level 229.83 mg g^−1^ of dry mass at 100 µm concentration. Reportedly, the asiaticoside yield was 5 times higher in the elicited cells than in the control of *C. asiatica* cultures [117]. Moreover, other concentrations of SA such as 50 μM and 150 μM have also shown positive effects on asiaticoside yield. Therefore, these results suggest to us that SA remarkably enhances the asiaticoside content in the suspension cultures of *C. asiatica*.

##### Methyl Jasmonate

Similarly, the effect of various concentrations of MeJA was observed on the triterpenoid content as well as hairy root growth of *C. asiatica*. It was reported that MeJA at higher concentrations causes browning in hairy roots which may be due to the release of phenolics, for example; in in vitro cultures of *C. asiatica*, the higher concentrations of MeJA have caused browning in hairy roots; however, at lower concentrations, it favored an increase in dry weight. Moreover, the triterpenoid content was also increased in the hairy root cultures of *C. asiatica* when the cultures were augmented with higher concentration when the medium was augmented with 400 μm MeJA. It has been further observed that with an increase in the concentration of MeJA, the triterpenoid concentration, especially madecassoside, also increased. Numerous other studies have reported that MeJA treatment to aerial parts and roots of *C. asiatica* significantly decreased the growth of the plant [118]. Sevón et al. [119] reported that by using MeJA as an elicitor for medicinal plants such as *Panax ginseng*, *Setaria parvifora* hairy roots, and *Eleutherococcus sessiliflorus* embryos, the biosynthesis of bioactive compounds significantly increased; however, the concentration, type of explant, and target secondary metabolites were different in each species. Therefore, the scientific community has resolved and reached a consensus that MeJA concentration for various plant species must be optimized [120]. Similarly, MeJA at 50 µM was observed to enhance the production of phenolic compounds in hairy root cultures of *Polygnum multiforum.* The elicitors act as signaling molecules recognized by the receptor proteins embedded in the plasma membrane and are believed to regulate the gene expression of secondary metabolites [121].

#### 4.2.2. Biotic Elicitors

##### Effect of Fungal Elicitors

Plant cell cultures are frequently being explored and investigated for various phytochemicals of higher medicinal value which are otherwise difficult to synthesize or produce from a native plant at insufficient concentration. The use of calli and liquid suspension cultures were more preferred because the desired metabolites can be easily targeted [122]. The effect of fungal elicitors on the production of biomass and asiaticoside were studied in shoot cultures of *C. asiatica.* It was observed that 3% culture filtrate of *Trichoderma harzianum* significantly enhanced the asiaticoside dry weight culture to 1.15 mg which is reported to be a 2.53- and 2.35-fold increase from the control or unelicited shoot cultures, particularly on the 10th day of 35 days of inoculation. Moreover, the biomass in the elicited shoot cultures also resulted in an increase in the biomass, reported to be a 1.24-fold increase as compared to the control. Similarly, the addition of 1.5% mycelial extract (ME) of the fungus *Colletotrichum lindemuthianum* on 0 day showed an increase in biomass accumulation; however, a decreasing trend in asiaticoside content (1.10 mg g^−1^ dry weight) was reported when compared to the control. Similarly, the treatment of *Fusarium oxysporum* in the range of 0.5–1.5% showed an inhibitory effect on the shoot growth when added in the medium on 0 day of the culture cycle with poor asiaticoside content with a GI (Growth Index) = 4.85–8.45 far less than the control 11.11 [60]. These results suggest that *Trichoderma harzianum* has a potential to enhance the secondary metabolites in *C. asiatica*, probably by influencing the biosynthetic pathway of triterpenes in *C. asiatica.* From amongst the various biotechnological approaches applied, the use of biotic and abiotic elicitors in cell and suspension cultures have shown promising results by initiating or activating the signal transduction pathways that possibly activate some promising genes involved in the biosynthetic mechanism of secondary metabolites [123]. It has been reported that type, duration, and concentration of an elicitor added in the medium has a significant effect on the production of secondary metabolites; however, growth stage, season, and type of plant also determine the outcome of an elicitation [109,124]. The multiple shoot cultures of *C. asiatica* were found to be more responsive to elicitation caused by the filtrates of *T. harzianum, F. oxysporum*, and *C. lindemuthianum.* The addition of the filtrate of *F. oxysporum* in the media, particularly at early stages of the culture, significantly inhibits the growth as well as asiaticoside accumulation as was observed for hyoscyamine and scopolamine synthesis in cell cultures of *Datura metel* [125]. This study demonstrated the potential of fungal elicitors to influence the biosynthetic pathways of secondary metabolites.

##### Bioconversion

The biotechnological advancements should aid in discovering alternatives for the production of medicinally important phytocompounds from plants without exploiting the natural population of the plant. The cell culture has descended into a new dawn and revolutionized plant studies by ensuring the plants are cultured or propagated on a large scale besides exploiting them for pharmaceutical compounds. Bioconversion is also one of the strategies of biotechnology that provides an alternative to enhance the bioactive compound of interest in higher quantity. The *Centella asiatica* cultures were supplied with precursors to bioconvert the α-amyrin into centellosides [126]. Reports suggested that α-amyrins are sold in the market at higher costs leading their accessibility in industrial markets to be almost negligible. Therefore, *C. asiatica* cultures were supplied with natural sources of precursors such as copal and MER to enhance the production of centellosides [126]. The *C. asiatica* cultures were supplemented with 50 mg/L α-amyrin, 250 mg/L copal resin, 125 mg/L Ext Copal, and 25 and 50 mg/L MER. The centelloside production was enhanced 70-fold in the cultures treated with α-amyrin and Ext Copal. It was further observed that after the 3rd day of treatment, the centelloside treatment was much higher than treatment on the 6th day. Moreover, copal resins were observed to cause death of the cells [126]. MER at 50 mg/L showed significant increase in the centellosides and achieved a yield similar to α-amyrin on day 3 after treatments.

##### Yeast Extract

Yeast extract was added to the cell cultures of *C. asiatica* at 2–5 g/L at the time of inoculation. It was observed that YE at various concentrations had a positive effect on both growth and production of asiaticoside. The positive effect on the growth of cell cultures of *C. asiatica* could be due to the ability of YE to act as a nitrogen source. It has to be noted that the asiaticoside content was lower than the cultures elicited with SA, perhaps due the growth effect of YE. Similarly, when 2–5 g of YE was added in the suspension culture of *C. asiatica*, the asiaticoside content enhanced from 61.38 mg g^−1^ of dry mass to 102.71 mg g^−1^ of dry mass [127].

### 4.3. Hairy Root Culture-Mediated Enhancement in Secondary Metabolites

The adventitious and hairy roots (HR) of a large number of medicinal plants are classified as a special plant parts or an outcome of biotechnological advancement that have the potential to harness phytocompounds in large numbers. HR cultures are produced by infecting the plant tissue with *Agrobacterium rhizogenesis* which allows its manipulated T-DNA from Ri-plasmid to integrate into the genome of a plant cell. The most important characteristic of T-DNA is the presence of genes that encode for auxin and cytokinin biosynthesis [128]. It was reported that the hairy roots of *C. asiatica* exhibit faster growth while maintaining biochemical and genetic stability [129]; however, the highest content of triterpenoids such as madecassoside and asiaticoside was obtained from in vitro and in vivo plants as reported by Aziz et al. [130]. Kim et al. [112] obtained 14.1% hairy roots in leaves and petiole of *C. asiatica* and increased the asiaticoside content by subjecting HRs to 100 µm methyl jasmonate [131]. Similarly, Alcalde et al. generated adventitious roots (AR) and HR cultures from leaf and petiole parts of *C. asiatica*. It was observed that all the HR lines derived from petiole leaf have shown higher levels of total triterpenoid content than all other transgenic lines, AD cultures, and nontransgenic lines of *C. asiatica*. Among various transgenic HR lines of *C. asiatica*, HR 2 lines have shown the highest triterpenoid content (46.57 mg∙g^−1^ dry weight [DW]), which increased 1.6-fold vs. the petiole-derived AR. Furthermore, madecassoside was detected in all the lines of HR and AR; however, asiaticoside was detected only in AR cultures derived from leaf and petiole explants of *C. asiatica.* Moreover, the secondary metabolites such as alkaloids, terpenoids and phenolics were observed to accumulate in transformed hairy root cultures as compared to callus cultures [132].

### 4.4. Precursor Feeding in Hairy Root Cultures of C. asiatica

Although cell suspension cultures positively influence the growth and enhancement of secondary metabolites, exogenous application of elicitors has shown significant results in increasing the phytocompounds of a plant [109]. Precursor feeding is an in vitro approach which involves the use of an intermediate phytocompound of a plant metabolism added in the medium to study the phytochemical analysis of culture cells. It has been exploited in the hairy root cultures of *Catharanthus roseus* for the enhancement of SM [114]. Squalene is an intermediate compound in the biosynthetic pathway of triterpenoids of *C. asiatica* [115]. Through mevalonate and deoxyxylulose phosphate pathways, four kinds of triterpenoids are biosynthesized, Figure 1. It has been reported that while using squalene as a precursor in the media, the hairy growth rate and triterpenoid content significantly increased. However, pyruvate as a precursor reduced the growth of hairy roots of *C. asiatica* [133]. However, both precursors were observed to enhance the content of triterpenoids in the hairy root cultures. Previously, Bohm and Mack [114] stated that precursors do not impact the growth of a culture as endogenous precursors are continuously being released by the metabolism. Sivanandhan et al. [116] reported that cell suspension cultures of *Withania somnifera,* when treated with squalene, significantly increased the biosynthesis of SM.

The development of an *Agrobacterium*-mediated transformation greatly influences the yield of biomass and secondary metabolites besides avoiding the disastrous effects of pesticides, heavy metals, and poor ecological conditions which otherwise negatively impact the cultivation [118]. Therefore, hairy root cultures promote fast growth in hormone-free media, molecular stability, and enhance the yield of secondary metabolites [119]. The effect of precursor feeding molecules such as squalene and pyruvic acid on the enhancement of SM in plant cell cultures has been reported in a number of plant species [120]. *C. asiatica* contains four important triterpenoids such as madecassoside, asiaticoside, madecassic acid, and asiatic acid, particularly in their leaf parts. To enhance the yield of these four compounds that are believed to have overarching medicinal properties such as anticancer, antidiabetic, wound-healing, anti-inflammatory, and neuroprotection ability [120], precursors such as squalene and pyruvic acid were applied to hairy root cultures of *C. asiatica* at varying concentrations and it was observed that squalene treatment greatly increased the triterpeniod contents of hairy root cultures and the highest triterpenoid content (57.53 mg g^−1^ DW) was reported to be 2.5 μM squalene, which is a 3.1-fold increase vs. control. Similarly, at 2.5 μM squalene treatment, the hairy roots showed an increase in the contents of asiaticoside and madecassoside, but at higher concentrations, 5 to 10 μM of squalene, the triterpenoid contents were significantly reduced. Similarly, in the hairy root cultures of *C. asiatica* when treated with pyruvic acid precursor, the triterpenoid contents increased 1.9-fold (26.28 mg g^−1^ DW) vs. control. Furthermore, at 5 μM pyruvic acid, the madecassoside contents significantly enhanced compared to that of the control; however, no such increase was observed in asiaticoside content. Overall, these results suggest to us that 2.5 μM squalene treatment significantly increases the triterpenoid content in the hairy root cultures of *C. asiatica* as compared to pyruvic acid precursor feeding treatment.

### 4.5. Transcriptomics Approach to Comprehend the Mechanism of Elicitation

Elicitation has become one of the frequent methods in manipulating the yield of secondary metabolites in *C. asiatica*. Considerable efforts have been made to enhance the production of triterpenoids in Centella by using both abiotic and biotic elicitors which has led to different results being achieved depending on the type of elicitors applied [92]. However, the most important aspect is to study the effect of a particular elicitor in the biosynthetic pathway of triterpenes. To comprehend the effect of elicitors on the biosynthesis of centellosides in *C. asiatica*, the expression of six genes involved in the biosynthesis of centellosides were analyzed by RT-qPCR in *C. asiatica* hairy root cultures subjected to elicitation. The samples were analyzed at 0, 8, 12, 24, 36 h, and 7 days. The study was conducted to shed light on the regulation of the biosynthetic pathway by elicitation. The elicitors used were methyl jasmonate and coronatine, added in both single and mixed form, and salicylic acid. The effect of the elicitors was mainly studied on four main phytocompounds of *C. asiatica*: that is, asiatic acid, madecassic acid, asiaticoside, and madecassoside. For the study of expression levels of the six genes, β-actin was used as a reference gene and untreated hairy root cultures were used as a control. The expression levels of genes in each treatment were compared with the control. It was reported that the expression levels of all the genes were higher when treated with either Coro or Coro + MeJA when observed at 8, 12, and 24 h after treatment. After 36 h of treatment, all the genes were upregulated, except the CYP716C11 gene. Similarly, all genes were upregulated at day 7, except CYP714E19, when hairy root cultures of *C. asiatica* were treated with Coro + MeJA. Similarly, MeJA treatment also showed significant levels of upregulation after 8 and 12 h of treatment; after that no difference in the expression levels of genes were analyzed between the control and the centelloside genes of the MeJA-treated hairy root cultures. SA elicitation induced a constant expression in all the samples [134].

The changes that are undergone at the transcriptional level can be helpful in determining the production of centellosides or mRNA. Transcriptomics is an emerging field in biotechnology that enables to determine the mRNA levels in a plant under a particular stress or condition. Motivated by this methodology, the gene expression levels of six centelloside genes were analyzed by qRT-PCR, and the data from transcriptomics and metabolomics was subjected to correlation to investigate the effect of an elicitor. SQS is one of the genes that is involved in the formation of precursor for triterpenoid and phytosterols [134]. It was reported that the SQS was upregulated in all the cultures treated with either Coro or Coro + Meja at 8 and 12 h after treatment. The subsequent decrease of SQS was attributed to its role in other key metabolic mechanisms [135]. Similarly, β-amyrin synthase (Figure 1) is involved in the synthesis of α- and β-amyrin (Figure 1), which act as precursors of various phytocompounds in *C. asiatica.* The expression of β-amyrin synthase in the hairy root cultures increased 400-fold after 12 h of treatment with Coro, and consistently remained high throughout the period of study when treated with Coro + Meja, slightly decreasing after the 7th day of treatment. Genes such as CYP716A83, CYP714E19, and CYP716C11 involved in the synthesis of saponins derived from α- and β-amyrin have shown a similar pattern of expression as that of β-amyrin synthase [134]. Similarly, Meja and salicylic acid have induced the highest expression levels of genes involved in the biosynthesis of centellosides; however, the most noticeable observations were achieved from the treatment of Coro and Coro + Meja at 8 and 12 after treatment. This study demonstrated that elicitation-caused upregulation can increase the production of centellosides in *C. asiatica.*

## 5. Conclusions

Medicinal plants have caught the attention of every member of society due to their growing demand among pharmaceutical, beverage, and cosmetic companies. *C. asiatica* is consumed in several countries and has been used against several diseases for centuries. Due to its great medicinal importance, the plant has been exploited to a very great extent. Moreover, no strategy has been formulated so far to conserve the natural population of the plant. Although efforts have been put forward to develop in vitro protocols for *C. asiatica*, only one or two studies have focused on biochemical and genetic stability of the in vitro raised plants of *C. asiatica.* Although, various approaches based on elicitation involving the use of both biotic and abiotic elicitors were employed to enhance medicinally important secondary metabolites such as asiatic acid, madecassic acid, and asiaticoside, the metabolomics approach is less touched upon by researchers which otherwise has a potential to enhance the metabolites of particular interest. Therefore, efforts should be made to focus on the enhancement of triterpenoids in order to meet the growing demand of pharmaceutical and cosmetic industries besides ensuring that no harm is done to the native population of the plant. In essence, this review aimed to deliver a summary of in vitro approaches and elicitation strategies for future aid in conserving and enhancing the triterpenoid content of the plant.

## Figures and Tables

**Table 1:** Phytocompounds of *Centella asiatica*.

Phytocompound Group	Phytocompounds	References
Terpenoids	Asiaticoside, triperpenes, centelloside, asiaticentoic acid, madecassic acid, betulic acid. Betacaryophyllene, germacrene, alpha-pinene	[12]
Phenols	Quercetin, rutin, naringin, castilliferol, kaempferol, luteolin, quercetin-3-o-β-d- glucuronide.	[12]
Phenols	Rosmarinic acid, 1,5-di-o-caffeoyl quinic acid, 4,5- di-o-caffeoyl quinic acid, 3,5- di-o-caffeoyl quinic acid,	[13]
Phenols	Tannin and phlobatannin.	[13]

**Table 2:** Biological activities of various phytocompounds of *Centella asiatica.*.

Phytocompound	Biological Activity	Name of the Disease	References
Madecassoside	Wound healing, anti-inflammatory, antiaging effects on skin and antipsoriasis effect. Downregulates the expression of important genes, COX-2 and PGE2, which maintain and aid in the progression of cancer. Inhibits the production of TNF-α. It treats rheumatoid arthritis which is a chronic inflammatory disease of joints usually observed in elderly people.	Headache, Alzheimer’s disease	[25,26]
Asiatic acid	Regulates various signaling pathways such as PI3K/AKT/mTOR and apoptotic pathways such as Bcl-2/Bax pathways. Protects mitochondrial activities. Inhibits the IL-1 beta-stimulated cardiomycete hypertrophic response. Improves cardiac function. Significantly improves lipid profile of plasma. Protects rats from undergoing diabetic nephropathy by inhibiting oxidative stress. Reduces O_2_^−^, MDA, nitrate level in plasma. Prevents kidneys from undergoing injury by suppressing proinflammatory cytokines NF-κB. Treats pelvic inflammation and ovarian cancer.	Stomach upset, gastrointestinal tract diseases	[27,28,29]
Asiaticoside	Inhibits apoptotic pathways. Has wound-healing properties. Promotes the synthesis of collagen. Enhances memory function. Reduces oxidative stress. Antidepressant activity. Downregulation of NF-κB signaling pathway which leads to inflammation. Suppresses cell invasion and migration.	Brain and nervous system disorders, neuroses, and depression	[30,31,32]
Quercetin	Antibacterial, antihepatotoxin, antiviral, antihypertensive, hepatoprotective, antimutagenic, diuretic.	Kidney, liver, viral diseases	[13,33,34,35]
Naringin	Anti-inflammatory, antiviral, cytotoxic, antioxidant, aldose reductase inhibitor.	Aging of the body, tumors	[36]
Apigenin	Aldose reductase inhibitor, diuretic, antihypersenstive, antiulcerative, and antioxidant.	Oxidative stress of the body, urine-excretory system	[37]
Kaempferol	Anti-HIV, anti-inflammatory, iodinate thyronine deiodinase inhibitor, and antioxidant.	Reduced immunity	[13]
Betulic acid	Cytotoxic, antibacterial, antineoplastic.	Bacterial diseases, tumors	[38]
Ascorbic acid	Antibacterial, antidote, antihypercholesterolemic, antioxidant, and inhibits the release of carcinogen.	Acute respiratory viral, bacterial diseases, oxidative stress	[13]
Chlorogenic acid	Cytotoxic, antiviral, choleretic, antimutagenic, and antimalarial.	Heals wounds, treats malaria	[13]
α- Pinene	Irritant, antitussive, and antifungal.	Fungal skin diseases	[39]
β –Pinene	Anti-inflammatory and antifungal.	Fungal skin diseases, decreased immunity	[39]

**Table 3:** Explant disinfection procedure in *C. asiatica*.

Explant	Sterilization Procedure	Reference
Leaf	Leaf → RTW → Liq. Extran 5% (5 min) → DDW → 0.1% HgCl_2_ (8–10 min) → 3 × DDW (5 min)	[57]
Nodes	Node → RTW (30 min) → Cetrimide 1% (150 mg/L bavistin + 50 mg/L trimethoprim) (25–30 min) → 0.1% HgCl_2_ (3–4 min) → 5 × DW	[58]
Nodes	Node → RTW → Liq. Labolein 5 to 10 drops/100 mL Teepol (5 min) → 1% Bavistin + 1% Blitox (15 min). → 0.1% HgCl_2_ (10 min) → 3 × DW	[59]
Shoot tip	Shoot tip → RTW (30 min) → Teepol (30 min)→ RTW 0.01% HgCl_2_ (5 min) → 3–4 × DDW	[62]
Nodes/Shoot tip	Nodes (30 min)→ 10% Teepol (15 min) → RTW (10 min) → 3 × DDW → 0.1% HgCl_2_ (8 min) → 3–6 × DDW	[63]
Axillary bud	Axillary bud → RTW (10 min) → 5% teepol (10 min) → RTW → 0.4% Bavistin → 70% Ethanol (60 s) → 0.1% HgCl_2_ (1–3 min) → 3–4 × DDW	[64]
Leaf/Stem	Leaf/stem → 2% Teepol → 0.1% HgCl_2_ (20 min) → 4–5 × DDW	[65]
Nodes	Nodes → RTW (30 min) → 5% Teepol (5 min) → DW → 0.1% HgCl_2_ (3 min) → 3–4 × DDW	[66]
Leaf/Stem	Leaf → RTW (30 min) → 2% liq. Detergent (10–15 min) → 1% Bavistin (1%) (1/2 h) → 3–4 × DW → 70% ethanol (30 s) → 0.1% HgCl_2_ (1 min) → 3–4 × DDW	[67]
Shoot tip	Shoot tip → RTW (15 min) → 2% teepol (10 min) → 70% ethanol (30 s) → 3–4 × DDW	[68]
Nodal explants	Node → RTW (50 min) → 2% teepol (5 min) → 0.1% HgCl_2_ (10 min) → SDW → 0.6% Bavistin + 0.05% Gentamycin (10 min) → SDW	[69]
Nodal segment	Nodes → RTW (30 min) → 1% teepol (5 min) → 0.1% HgCl_2_ + teepol (3 min) → 3–4 × SDW → 0.1% HgCl_2_ (3 min) → SDW	[70]
Shoot tip	Shoot tip → RTW (30 min) → 1% tween 80 (10 min) → SDW → 0.1% HgCl_2_ (10 min) SDW (repeated times)	[71]

DDW, double distilled water; DW, distilled water; HgCl_2_, mercuric chloride; NaOCl, sodium hypochlorite; RTW, running tap water; EtOH, ethyl alcohol (ethanol); s, second(s); minute (min); SDW, sterilized distilled water; SW, sterilized water; H_2_O_2_, hydrogen peroxide.

**Table 4:** Direct organogenesis in *C. asciatica*.

Explant	Treatment	Culture Conditions	Number of Shoots, Pcs	References
Nodal segment	MS + BAP (2.2–44.4 μM) + NAA (0.00–5.37 μM) MS + BAP (2.2–8.9 μM) + IAA (2.88 μM)	pH = 5.8, sucrose = 3%, Agar 0.8%, Temp = 24 ± 2 °C, Light = 16 h photoperiod of 50 µmol m^−2^s^−1^	6–7	[59]
Nodal segment	MS + BAP (1–5 mg/L) + IBA or NAA (0.5 or 0.25 mg/L) MS + BAP (4.0 mg/L) + NAA (0.1–0.5 mg/L)	pH = 5.8, sucrose = 3%, Agar 0.8%, Temp = 24 ± 2 °C, Light = 16-h photoperiod of 40 µmol m^−2^s^−1^	7–8	[59]
Shoot tip	MS + BA (0.1–2.5) + NAA or KN (0.1–1 mg/L)	pH = 5.8, Sucrose = 30 g/L, Temp = 25 ± 3 °C, Light = 16 h photoperiod of 54 µmol m^−2^ s^−1^	6–8	[60]
Shoot tip/nodal segment	MS + BAP (2.0 mg/L) + KN (0.5 mg/L)	pH = 5.8, Sucrose = 3%, Agar = 0.8%, Temp = 25 ± 2 °C under 16 h light intensity of 3000 lux	6–8	[63]
Nodal explants	MS + BAP (1.5 mg/L) + KN (1.0 mg/L)	pH = 5.8, Sucrose = 3%, Agar = 0.8%, Temp = 25 ± 2 °C under 16 h light intensity of 3000 lux	6–8	[64]
Nodal explants	MS + BAP (2.0 mg/L) + Bavistin (150 mg/L)	pH = 5.8, Sucrose = 3%, Agar = 0.8%, Temp = 26 ± 2 °C, Light = 16 h light intensity of 3000 lux	7–8	[72]
Shoot tip	MS + BAP (17.76 μM) + GA_3_ (1.44 μM)	pH = 5.8–6.0, Sucrose = 3%, 0.8% agar, Temp = 24 ± 2 °C, Light = 16 h light intensity of 60 µE/m^2^ s)	6–7	[68]
Nodal explants	MS + BAP (4.0 mg/L) + NAA (0.5 mg/L)	pH = 5.8, Sucrose = 3%, Agar = 0.8%, Temp = 26 ± 1 °C, Light = 16/8 light and dark cycle with 3000 lux intensity	10–12	[69]
Nodal segment	½ MS + BA (0.2 mg/L) alone or in combination with NAA and IAA (0.5 mg/L)	pH = 5.6–5.8, Sucrose = 3%, 0.7% agar, Temp = 24 ± 2 °C, Light = 12 h photoperiod with light intensity 35–40 µmol m^−2^ s^−1^	10–13	[70]
Shoot tip	MS + BAP (4.0 mg/L) + NAA (0.1 mg/L)	pH = 5.8, Sucrose 3%, Agar = 0.8%, Temp = 25 ± 2 °C, Light = 16 h photoperiod at 3000 lux light intensity	6–8	[71]

BAP, 6-Benzylaminopurine; BA, benzyl adenine; KN, kinetin; IBA, indole-3-yl-butyric acid; NAA, α-Naphthalene acetic acid; IAA, indole acetic acid; GA_3_, gibberellic acid; cm, centimeter; mg/l, milligram per litre; h, hour; µM, micromolar; SPFD, spectral photon flux density; CWFT, cool white fluorescent tube; PP, photoperiod; RH, relative humidity; Temp., temperature.

**Table 5:** Indirect organogenesis in *C. asciatica*.

Explant	Treatment	Culture Condition	Remarks	References
Internode	MS + NAA (5.37 μM) + KN (2.32 Mm) (ECIM). MS + NAA (2.69 μM) + KN (1.16 μM) (SEMM)	Sucrose = 3%, Agar = 0.7%, pH = 5.8. Temp = 25 ± 2 °C. Photoperiod = 16/8 h 25 mmol m^−2^ s^−1^ provided by CWFT.	Total 204.3 mean number SE produced per 100 mg of callus. In SEMM, 88% the embryos matured and converted to plantlet. Additionally, embryo-derived plantlets are morphologically identical to parent plant.	[57]
Leaves	MS + 2,4-D (2.0 mg/L) MS + 2,4-D (2.0 mg/L). MS (devoid of hormone) Embryo culture after every 4 weeks.	Sucrose = 3%, Agar = 0.7%, pH = 5.8. Temp= 25 ± 2 °C. Photoperiod = 14 h provided by CWFT.	SE were induced from the leaf explants when cultured on MS basal medium supplemented with 2,4-D. Onset of nodular callus and development of SEs was observed on explants after 3 to 4 weeks of culturing in the dark.	[84]
Leaf/Internode	MS + 2,4-D (2.0 mg/L) + KN (0.5 mg/L) (CIM). MS + 2,4-D (0.5 mg/L) + KN (0.25 mg/L) (SIM).	Sucrose = 3%, Agar = 0.7%, pH =5.8. Temp = 25 ± 2 °C. Photoperiod = 14 h provided by CWFT.	On callus induction medium, creamy friable calli were developed. Subculturing of these calli on lower concentration of 2,4-D and KN started developing globular embryo after 30 days. Using ½ strength MS medium devoid of any growth regulators helped in the maturation of somatic embryos. The shoot pole of the embryo remained dormant and clustered and the root pole showed normal development. However, the embryo formed did not germinate.	[85]
Nodal	MS + BAP (2.0 mg/L) + NAA (0.2 mg/L) (ECIM). MS + BAP (2.0 mg/L) + NAA (0.2 mg/L) (SEIM).	Sucrose = 3%, Agar = 0.7% pH = 5.8. Temp = 25 ± 2 °C. Photoperiod = 14 h provided by CWFT.	Higher percentage of embryogenic callus was reported on ECIM. The embryogenic callus produced 246.66 mean number of somatic embryo. Out of total embryo produced, 40% showed in vitro germination. Germinated embryos were successfully converted into plantlets with 80% survival rate.	[85]

MS = Murashige and skoog medium; ECIM, embryogenic callus induction medium; SEMM, somatic embryo multiplication medium; SEGM, somatic embryo germination medium; CM, coconut milk; M, molar/BAP = 6-Benzylamino purine, 2,4-D, ZT, zeati; d, days; 2,4-D, 2,4-dichloro phenoxyacetic acid; BAP, 6-Benzylaminopurine; Ba, benzyl adenine; TDZ, thidiazuron; KN, kinetin; IBA, indole-3-yl-butyric acid IBA; NAA, α-Naphthalene acetic acid; cm, centimeter; no, number; h, hour; wk, weeks; mg, milligram; L, liter; mmol, millimolar; µM, micromolar; CIM, callus induction medium; SIM, shoot induction medium; SEIM, somatic embryogenic induction medium.

**Table 6:** In vitro rooting in *C. asciatica*.

Treatment	Mean Root Number	Root Length	Planting Substrate	Plant Survival, %	References
½ MS + IBA (0.49–2.46 μM)	8.5	6.3 cm	Garden soil or soilrite	90	[58]
MS + BAP (5 mg/L) + IBA (0.5 mg/L)	2.0	NR	Vermiculite soil, sand, and FYM (farmyard manure) in 1:1:1 ratio	90	[59]
MS + IBA (1.0–3.0 mg/L) and NAA (0.5–2.0 mg/L)	46.8	19.7 cm	Vermiculite soil: sand (1:1) mixture	98–99	[62]
MS + NAA (1.0 mg/L) + IBA (1.0 mg/L)	16.5	NR	Sand, soil, and farmyard manure (1:1:1)	80	[63]
MS + IBA (2.0 mg/L)	NR	NR	NR	NR	[64]
MS + IBA (2.0 mg/L)	14.2	4.2 cm	Soil and vermiculite in 1:1	90	[72]
½ MS + NAA (10.74 μM)	27.66	4.90 cm	Red soil and vermiculite	95	[68]
MS + IBA (0.5 mg/L)	4.5	NR	-	NR	[69]
MS + IBA (1.0 mg/L)	10.6	4.30 cm	Sand, soil, and farmyard manure (1:1:1)	80	[70,71]

NR, not reported; BAP, 6-Benzylaminopurine; IBA, indole-3-yl-butyric acid; NAA, α-Naphthalene acetic acid; cm, centimeter; µM, micromolar; mg/L, milligram per liter.

## Data Availability

Not applicable.
